# Toward a New Paradigm of North–South and South–South Partnerships for Pandemic Preparedness: Lessons Learned from COVID-19 and Other Outbreaks

**DOI:** 10.4269/ajtmh.22-0466

**Published:** 2022-11-14

**Authors:** Sabin Nsanzimana, Angeli Rawat, Lindsay A. Wilson, Jamie I. Forrest, Gilmar Reis, Sreeram Ramagopalan, Jean-Jacques Muyembe-Tamfum, Francine Ntoumi, Alimuddin Zumla, Papa Salif Sow, Jean B. Nachega, Agnes Binagwaho, Mark Dybul, Edward J. Mills

**Affiliations:** ^1^ University Teaching Hospital, Butare, Rwanda;; ^2^ University of Global Health Equity, Rwanda;; ^3^ Department of Health Research Methods, Evidence and Impact, Faculty of Health Sciences, McMaster University, Hamilton, Canada;; ^4^ University of British Columbia, Vancouver, Canada;; ^5^ Platform Life Sciences, Vancouver, Canada;; ^6^ Department of Real World and Advanced Analytics, Vancouver, Cytel, Vancouver, Canada;; ^7^ Pontifícia Universidade Católica de Minas Gerais, Belo Horizonte, Brazil;; ^8^ Roche, Basel, Switzerland;; ^9^ Institut National de la Recherche Bio-Médicale (INRB), Kinshasa, Democratic Republic of the Congo;; ^10^ Fondation Congolaise pour la Recherche Médicale, Brazzaville, Republic of Congo;; ^11^ Institute for Tropical Medicine, University of Tübingen, Tübingen, Germany;; ^12^ Division of Infection and Immunity, Centre for Clinical Microbiology, University College London;; ^13^ NIHR Biomedical Research Centre, University College London Hospitals NHS Foundation Trust, London, United Kingdom;; ^14^ Faculte de Medecine Service des Maladies Infectieuses, Universite Cheikh Anta Diop Dakar, Senegal;; ^15^ Department of Medicine, Division of Infectious Diseases, Stellenbosch University Faculty of Medicine and Health Sciences, Cape Town, South Africa;; ^16^ Departments of Epidemiology and International Health, Johns Hopkins Bloomberg School of Public Health, Baltimore, Maryland;; ^17^ Departments of Epidemiology, Infectious Diseases and Microbiology, University of Pittsburgh School of Public Health, Pittsburgh, Pennsylvania;; ^18^ University of Global Health Equity, Kigali, Rwanda;; ^19^ Center for Global Health Practice and Impact, Georgetown University, Washington, DC

## Abstract

COVID-19 underscores the need to reimagine North–South partnerships and redefine best practices for building public health and research capacity to address emergent health threats and pandemic preparedness in low- and-middle income countries (LMICs). Historically, outbreak and emergency responses have failed to ensure that the Global South has the autonomy and capacity to respond to public health threats in a timely and equitable manner. The COVID-19 response, however, has demonstrated that innovations and solutions in the Global South can not only fill resource and capacity gaps in LMICs but can also provide solutions to challenges globally. These innovations offer valuable lessons about strengthening local manufacturing capacity to produce essential diagnostic, treatment, and prevention tools; implementing high-quality research studies; expanding laboratory and research capacity; and promoting effective cooperation and governance. We discuss specific examples of capacity-building from Rwanda, South Africa, and Senegal. To fulfill promises made to the Global South during the COVID-19 pandemic, restore and resume health service delivery, and effectively prevent and respond to the next health threat, we need to prioritize equitable access to local manufacturing of basic health tools while building health systems capacities in the Global South.

The COVID-19 pandemic has highlighted health disparities between the Global South and the Global North and exacerbated existing challenges in clinical research and the equitable provision of vaccines, diagnostics, and therapeutics. Although many factors contributed to the differences in COVID-19 mortality and morbidity rates between countries,^1^ the pandemic has underscored the urgent need to reimagine how evidence is generated and used between the Global North and South.

Locally led research supports local ownership and buy-in, ensures research is culturally relevant, and promotes sustainability while simultaneously strengthening local capacity.^2^ However, clinical trial funding and research capacity has historically flowed from the Global North to the Global South, thus maintaining power imbalances between high- and low-income countries^3^ and eroding local capacity to lead research and evidence mobilization.^4^^,^^5^ With the increasing recognition that resilience is an essential property of a health system when shocked or stressed,^6^^,^^7^ building capacity for countries in the Global South to respond to their own needs has never been more important.

The need to reimagine North–South partnerships was highlighted during the COVID-19 response, when global supply chains were interrupted and health systems’ capacities were limited by shortages of material and human resources. Recognizing the need to address the health challenges arising from COVID-19, countries in the Global South drew upon local capacity to develop their own innovative solutions (Boxes 1–3). Several lessons can be learned from these solutions and hold tremendous potential for capacity-building and pandemic preparedness around the world.

Box 1 **Lessons learned from Rwanda**

Rwanda’s preparedness and infrastructure for responding to infectious diseases such as HIV infection, malaria, tuberculosis, and Ebola bolstered their COVID-19 response. Ebola preparedness measures (e.g., temperature screening, sanitation protocols, robust surveillance systems) were easily adapted to COVID-19. Furthermore, the government built upon preestablished public trust in vaccination programs to encourage uptake of COVID-19 vaccines^17^ and invested in data systems to capture real-time information at community and health facility levels. Electronic platforms permitted access to testing and vaccination status, allowing for quick decision-making regarding COVID-19 control measures. Surveillance systems were easily modified to identify potential cases, and rapid response teams conducted investigations to identify possible exposures and risk factors. All these efforts helped to ensure a coordinated and effective COVID-19 response.

Rwanda also experienced extreme shortages of PPE and therapeutics during the COVID-19 response. With the purchase of necessary equipment and incentives for local manufacturing companies, Rwanda invested in brand-new local manufacturing capacities for PPE, thereby generating enough PPE for the country and creating a sustainable long-term supply chain. Rwanda also leveraged diplomatic channels to assist with procurement. Diplomats and delegates visited factories in India, China, and Belgium and forged streamlined procurement protocols to assist in future responses, highlighting the importance of diversifying procurement partners and stockpiling essentials to build resilience.

Furthermore, many countries in Africa were unable to maximize their use of COVID-19 vaccines due to a lack of cold storage capacity. Rwanda was the first country in Africa to deploy the vaccine, thanks to proactive planning and preparedness. While microRNA vaccines were still in development, the country purchased deep freezers to store the incoming vaccines. Rwandan stakeholders learned from their experiences with PPE shortages to anticipate their next need and made a contingency plan accordingly.

Finally, as in many other parts of the world, diagnostic tests for COVID-19 were in short supply in Rwanda. To address this gap, Rwandan scientists repurposed disease-specific testing platforms to be used for COVID-19 and developed a method of pooled testing where 20 samples could be tested at once.^18^ This strategy has since been adopted in many countries worldwide, highlighting the importance of bidirectional knowledge transfer that is mutually beneficial.

Box 2 **Lessons learned from Senegal**

Senegal dramatically scaled up its testing capacity and was one of the first countries on the African continent to test for COVID-19. Using a decentralized strategy that provided results via a digital platform and streamlined laboratory operations, test results were available in less than 24 hours.^19^ The Senegalese developers at the Pasteur Institute of Dakar have partnered with the United Kingdom to produce a COVID-19 test that does not require any laboratory capacity and provides results in 10 minutes. This first-of-a-kind partnership will build capacity in Africa and promote access for COVID-19 diagnostics across the continent to ensure COVID-19 tests are available at cost.

In addition, Senegal’s Pasteur Institute of Dakar is currently producing a prequalified yellow fever vaccine and has recently reached a deal with the U.S. company MedInstill for the preparation of doses of COVID-19 vaccine. The institute is also working with BioNtech, along with Rwanda and Ghana, to start local production of mRNA vaccines in 2022.

Box 3 **Lessons learned from South Africa**

South Africa has contributed 41% of the African SARS-CoV-2 genomic sequences through its expanded national network of laboratories. South Africa and Botswana’s capacities to sequence and identify the B.1.1.529 (Omicron) variant was critical for disseminating evidence globally and identifying potential treatments. This sequencing triggered scientific debate on the best way to treat Omicron variant infections, as previously identified therapies such as monoclonal antibodies appeared not to be effective on this variant.^20^ However, the region’s scientific contributions quickly turned into retaliatory travel bans, a consequence that may serve as a disincentive for countries to share sequences in the future.^21^ These lessons further highlight the need for international data sharing without consequence and for countries to be able to respond to current shocks without risking gains made through their health systems.

## BUILDING LOCAL AND REGIONAL MANUFACTURING CAPACITY

A major consideration that has emerged from the COVID-19 pandemic is the need to strengthen local and regional manufacturing of essential health tools such as personal protective equipment (PPE), vaccines, diagnostics, and therapeutics. Historically, China, India, and Global North–based manufacturers have provided these products to other countries, but supply chain disruptions during the COVID-19 response led to global shortages. Shortages were especially severe in countries without the purchasing power of the Global North, including many countries in Africa.

Several initiatives on the African continent sought to improve access to such tools, including the Africa Medical Supplies Platform, a not-for-profit initiative developed by the African Union and its Center for Disease Prevention and Control. These partnerships enabled immediate access to efficient and reliable manufacturers and procurement partners by pooling resources to increase the purchasing power of African countries. This allowed for the purchase of certified medical equipment and facilitated equitable access to, as well as payment, logistics, and transportation for, critical supplies for countries most in need. Additionally, local manufacturing of PPE and test kits in the Global South enabled local access to these essentials. Extending this manufacturing capability to include medicines is a logical next step in the Global South but will require commitment from drug and device manufacturers in the Global North to facilitate knowledge transfer. Given the challenges associated with the rollout of COVID-19 vaccines in the Global South, this cooperation may be a significant obstacle.

In addition to inequities in access to essential supplies, vaccine inequity has been one of the greatest failings of the global COVID-19 response.^8^ The African continent currently produces only 1% of the vaccines it uses,^9^ fueling dependency on vaccines from higher-income countries. These dependencies and inequities appeared particularly stark when, during the emergence of the SARS-CoV-2 Delta variant, India suspended many of its exports, including prepaid shipments of COVID-19 vaccines. Leaders on the African continent have pledged to increase the share of vaccines manufactured in Africa from 1% to 60% by 2040^9^ by partnering with vaccine manufacturers to build factories and provide skills and knowledge transfer. Initiatives that aim to build local vaccine production include the shipment of 800 square meters of BioNTech-designed bio-containers to Senegal, Ghana, and Rwanda by the end of 2022^10^ and the building of a Moderna mRNA vaccine manufacturing facility in Kenya by 2023.^11^ These initiatives, however, can be effective only when intellectual property is openly shared in a timely manner. Commitments to eliminate legal barriers to equitable access across governments, pharmaceutical companies and policymakers must be made and honored.

## BUILDING OWNERSHIP IN SURVEILLANCE, DATA MANAGEMENT, AND RESEARCH

The pandemic has also demonstrated the importance of high-quality surveillance, data management, and research capacity that is owned by institutions in the Global South. Infrastructure that can be adapted quickly is critical to an effective emergency response. However, in some countries, COVID-19-related surveillance and infrastructure were managed by vertical programs that were designed in crisis mode and were often inefficient for maximizing scarce resources or quickly deploying mitigation strategies. The impact of diverting resources away from social programs to fund pandemic responses was felt around the world, but the impacts of such diversions were disproportionately severe in the Global South. As the COVID-19 pandemic wore on, these emergency programs became unsustainable and affected the control of other diseases, thereby risking hard-won gains in the management of these diseases.^12^^,^^13^

In the current era of decolonizing global health, more inclusive participation is needed in research and in the generation and analysis of health metrics. There is an opportunity for the Global South to take a leadership role in real-time data collection and monitoring. By strengthening local surveillance systems, researchers in the Global South can contribute to locally relevant pandemic preparedness and modeling and move away from the structural inequities inherent in the current management of global health threats.^14^ By building and strengthening digital health data platforms, countries in the Global South can improve disease surveillance and management. Furthermore, researchers should be incentivized to combat brain-drain and ensure research is a sustainable career that promotes innovative local solutions to health challenges. Investment in education and training for healthcare workers and nonclinical staff will be an essential component of supporting the next generation of global health experts.

The establishment of the pan-African multidisciplinary COVID-19 research consortium by the African Forum for Research and Education in Health (AFREhealth) provides a compelling illustration of the benefits of using real-world evidence to generate valuable global health insights. AFREHealth addresses research questions about COVID-19 infection in the African context by pooling routinely collected data from multiple countries to inform health policy and practice across sub-Saharan Africa. These context-specific data are essential for informing clinical practice in Africa,^15^ but the varying quality and quantity of data from across the Global South means that important knowledge gaps remain. Uniformly collected, high-quality data are needed to guide the development of reliable metrics for health systems and pandemic preparedness and response.^14^

## STRENGTHENING LOCAL CAPACITIES FOR CLINICAL RESEARCH

Currently, research and trials conducted in the Global South are often led by scientists from the Global North, and the results of these trials are often difficult to access by decision-makers in low-income settings. These challenges underscore the need to strengthen research capacity in the Global South by investing in people and partnerships to produce the evidence required to combat current and future threats. In particular, the emergence of diseases such as the Ebola virus disease, monkeypox, and COVID-19 have highlighted the need for future capacity-building efforts to leverage preparedness initiatives working at the human–animal–environment disease interface.^16^

## THE NEED FOR SHARED COOPERATION AND GOVERNANCE

In addition to the need for strengthened local research capacity within countries in the Global South, greater trust and collaboration are needed between nations and stakeholders to promote South–South partnerships that can ensure coordinated data sharing, vaccine and therapeutic development, and equitable access to treatments. These efforts should be proactively initiated and funded to take advantage of lessons learned during the current health crisis. The Global South’s dependency on resources and data from the Global North should continue to be reduced, and knowledge exchange channels should be strengthened. Bureaucratic procurement processes were streamlined during the pandemic, and similar opportunities to harmonize partnerships and processes should continue to be evaluated. The COVID-19 pandemic has demonstrated that countries need to work together—not as a choice or a privilege of the rich to help the poor, but as a common obligation. In our globalized world, strengthened research capacity and partnerships are to everyone’s benefit because disruptions in international cooperation contribute to a health threat’s global toll. Global coordination and cooperation will be critical to recovery from this pandemic and to ensuring global preparedness in the future.

## CONCLUSIONS

Although international and local partnerships play a critical role in global health, the COVID-19 experience has highlighted the need to reimagine these partnerships. There is an urgent need to invest in clinical research and train and retain skilled personnel in locations with the highest disease burden. Collaborations should seek to address barriers to an effective global health response while also promoting self-reliance and access to therapeutics, diagnostics, vaccines, equipment, and other technologies. A global response can only be as effective as the strength of the health systems and partnerships within them. Continuing to build health systems’ capacities in the Global South will help the global community as a whole respond to any shock that may come next.
